# Universal Screening in Positive School Mental Health Using the ASEBA Methodology for Teachers: A Pilot Epidemiological Study

**DOI:** 10.3390/ijerph182211807

**Published:** 2021-11-11

**Authors:** Antonio Cortés-Ramos, Miguel Landa-Blanco

**Affiliations:** 1 Department of Developmental Psychology and Education, Faculty of Psychology and Speech Therapy, University of Malaga, 29010 Malaga, Spain; 2 Degree in Clinical Psychology, School of Psychological Sciences, National Autonomous University of Honduras, Tegucigalpa 11101, Honduras; miguel.landa@unah.edu.hn

**Keywords:** universal screening school mental health, ASEBA methodology, positive school mental health, school mental health promotion, early prevention school mental health

## Abstract

School-based detection and intervention are critical components in ensuring positive mental health in children, with teachers playing an essential role in assessing students’ well-being. The current research aims to be a pilot epidemiological study on positive school mental health in Malaga, Spain, using the Achenbach System of Empirically Based Assessment (ASEBA). Data were collected in the COVID-19 pre-pandemic setting, using the Caregiver-Teacher Report Form (C-TRF) and the Teacher Report Form (TRF) in a sample of 420 children, who were between 5 and 8 years old at the time of the data collection. In 5-year-old children, the DSM-oriented scale with the highest clinical prevalence corresponds to attention deficit and hyperactivity problems (1.13%). In this same sub-sample, clinical levels of externalizing problems (4.52%) were non-significantly more common than internalizing conditions (1.69%). As for children between 6 and 8 years old, the DSM-oriented scale with the highest prevalence of clinical scores corresponds to anxiety problems (4.12%) and conduct problems (2.88%). Clinical levels of externalizing problems (9.47%) were non-significantly more prevalent than internalizing problems (6.58%). The results present 95% confidence intervals prevalence data in the general population and sex-differentiated descriptive statistics. The results are discussed according to their implication for school mental health.

## 1. Introduction

### 1.1. The Sustainable Development Goals and Positive School Mental Health

The United Nations (UN) has identified 17 Sustainable Development Goals (SDGs) that are urgent and necessary to achieve peace and prosperity worldwide. Such SDGs focus on various themes, including economic development, equality, health, and education. Specifically, SDG-3 and SDG-4 are targeted at good health and well-being and quality education, respectively [1].

Health, in general, and mental health, in particular, are critical components of the SDGs. The World Health Organization (WHO) defines health as a biopsychosocial state of well-being beyond the absence of disease [2]. It is within this framework that the importance of mental health in the educational system is evident. In this sense, there is no health without mental health [3]. Mental health is a core element in the development of the SDGs as it influences sustainability and social and economic development. In turn, economic and social development are variables that determine the mental health of individuals, particularly in the first years of life [4,5,6].

The SDGs recognize children as agents of change, being the central axis of the development of our societies. Thus, it is essential to improve children’s well-being in order to achieve sustainable and equitable development. The 2030 Agenda aims to reach all children, especially the most vulnerable [7], to develop the necessary intellectual abilities, creative thinking, and well-being to become healthy and productive adults [8].

### 1.2. The Importance of School-Based Mental Health Screenings and Interventions

Early childhood development is of extreme importance. Since birth, children may experience the accumulation of adversities that may have a lasting negative impact on mental health, learning skills, inequality, stigmatization, and social exclusion [8,9,10,11,12]. 

In addition to being constituted as a formal learning context, school is directly linked to positive mental health. It promotes children’s development by reducing stigmatization, strengthening academic skills and achievements, well-being, equal opportunities, and inclusion [13,14,15]. Therefore, the school setting constitutes a vital platform for mental health prevention and promotion [16]. Especially when considering the time that students spend within the school setting during their most important years of development and the fact that schools can simultaneously reach a large number of children [8,17,18]. 

Because of this, schools systems can be used as a platform to detect and intervene in children’s mental health issues [16]; such data is helpful in the design of public policies [19]. However, although most students could benefit from poor mental health prevention programs, not all receive the necessary psychological support. There is no early identification of at-risk students, producing an undervaluation and under-detection of students with mental health problems [20,21]. 

Health-promoting schools constantly reinforce their capacity as healthy places to live, learn, and work [22]. To achieve this, it is necessary to involve teachers, students, parents, and the community. Currently, schools work under a psycho-pedagogical model, which focuses on students’ academic achievement, not addressing aspects related to the prevention and promotion of universal school mental health. The protocol for detection, identification of students with specific educational support needs, and organization of the educational response of the Counseling of Education of the Andalusian Board are as follows [23]: The teaching staff identifies signs of specific educational support needs; meetings are made with both teachers and family members to determine educational intervention to optimize students’ academic achievement. If the difficulties persist after applying the intervention in no less than three months, a request for psycho-pedagogical evaluation is made. The tutor submits the request to the school authorities. The counselor analyzes the interventions made and decides whether to proceed with a psycho-pedagogical evaluation. If the evaluation of the student proceeds, the tutor summons the parents or legal guardians to authorize such an assessment.

### 1.3. The Achenbach System of Empirically Based Assessment

The Achenbach System of Empirically Based Assessment (ASEBA) has been widely used as a screening mental health tool in school settings [24,25,26]. The present study will focus on the Caregiver-Teacher Report Form (C-TRF) and the Teacher Report Form (TRF). Both of which assesses syndromes (internalizing and externalizing problems) and DSM-oriented disorders. The C-TRF is designed to assess children 1.5–5 years old; it has scales based on the DSM. These include affective, anxiety, pervasive developmental, attention-deficit/hyperactivity, and oppositional defiant problems. A similar structure is kept for the TRF, applied to children 6–18 years old; however, pervasive developmental problems are substituted by somatic problems. 

According to the ASEBA, symptoms can be classified as internalizing or externalizing syndromes. Internalizing disorders include symptoms of anxiety, depression, withdrawal, and somatic complaints. Anxiety is the anticipatory psychophysiological response to future threats; it is often characterized by muscle tension, hypervigilance, and avoidant behaviors. Children may develop anxiety disorders that, without proper intervention, may persist through adulthood. On the other hand, depressive symptoms often include feelings of sadness, emptiness, and irritability [27]. Compared to adults, symptoms of depression in children may be more characterized by irritability than by low mood. Depression may deteriorate children’s functionality in home and school settings [28].

Additionally, somatic problems include aches, headaches, stomachaches, nausea, skin, and eye problems [25,26]. Externalizing problems include rule-breaking and aggressive behaviors [25,26], negatively affecting children and adolescents’ school life. In this regard, there is evidence of a significant inverse relationship between externalizing problems and educational success [29]. It is important to notice that internalizing and externalizing problems are not mutually exclusive and may be correlated with each other [30]. A study conducted in Finland measured internalizing and externalizing symptoms of 8-year-old children and then repeated measures to those same cases at age. The results indicated that teachers’ baseline reports on conduct problems were significant predictors of externalizing problems later in life [31].

### 1.4. Prevalence of Internalizing and Externalizing Problems in Children

A meta-analysis based on children’s mental health in 21 countries determined that mental disorders had an overall prevalence of 13.4%. Common disorders included anxiety (6.5%), disruptive disorders (5.7%), attention deficit and hyperactivity disorders (ADHD) (3.4%), and depressive disorders (2.6%) [32]. 

A study carried out among 1286 0 to 14-year-old children in Spain determined that psychiatric pathologies were found in 11.5% of the selected sample. The most common disorders included ADHD (5.36%) and language disorders (3.42%), 3.26% of the sample had learning disorders, 2.4% had anxiety-depressive disorders, and 1.87% had disruptive disorders [33]. Other studies have found the prevalence of anxiety disorders to be between 2.4% and 13.3%, and depressive disorders between 3% and 14.6%. Behavioral problems show a prevalence ranging between 20.1% and 23.0%; while the prevalence of ADHD varies between 4.2% and 10.9% [33,34,35,36,37].

### 1.5. The Present Study

Considering the above, the current research aims to be a pilot epidemiological study on positive school mental health in Malaga, Spain, using the ASEBA methodology for teachers. Specifically, the study uses the C-TRF for 5-year-old children and the TRF for children between 6 and 9 years old. Prevalence data are presented for the general population, as well as separately for boys and girls. It is worth noting that this research is part of a more extensive study that started in 2016, and it is still ongoing. It is important to indicate that the current dataset was processed in a pre-pandemic setting (2016–2019).

In the long run, the results of our study can be used as a foundation to transform an inequality-generating model into a universal model of prevention and promotion of school mental health from which all students benefit [38]. Our study is extremely relevant to universal school mental health programs, given the importance of early detection and intervention. 

## 2. Materials and Methods

### 2.1. Participants

At the time of this study, the number of children (i.e., 5–8 years old) attending public schools in Fuengirola, Malaga, was 2810 children. Of which 51% (*N* = 1447) were boys and 49% (*N* = 1363) girls. Using a 95% confidence level and a 5% margin of error, the minimum required sample size was 339 cases. The sample selection process followed a non-probabilistic approach, in which the selected sample only included children whose parents gave written informed consent to participate in the study. The final sample size was 420 children, a number well above the required minimum stated originally. Of those, 42.14% (*n* = 177) used the C-TRF and 57.86% (*n* = 243) used the TRF. The total average age was of 5.90 years (*SD* = 0.92); for the C-TRF sub-sample, the mean age was of 5 years (*SD* = 0.00), while for the TRF sub-sample, the mean age was of 6.56 years (*SD* = 0.655). A full characterization of the sample is presented in Table 1. 

### 2.2. Instruments

#### 2.2.1. The Caregiver-Teacher Report Form (C-TRF)

The Caregiver-Teacher Report Form (C-TRF) is a 100-item instrument that evaluates two different scales: the DSM-oriented scale and the Syndrome scale. The DSM-oriented scale consists of: affective problems, anxiety problems, pervasive developmental problems, attention deficit and hyperactivity problems, and oppositional defiant problems; their internal consistency scores range from 0.65 to 0.92 On the other hand, the Syndrome scale evaluates both internalizing (emotionally reactive problems, anxious and depressed problems, somatic complaints and withdrawal) and externalizing problems (attention problems and aggressive behaviors). The internal consistency scores of the Syndrome scales range from 0.65 to 0.94, see Table 2.

The teacher scores each item from 0 to 2 points, were: 0 = “not true”, 1 = “sometimes true”, and 2 = “very true”; higher scores indicate a higher symptom intensity. Raw scores are then converted to *t*-scores, which are classified as: normal (*t* < 65), risk (65 < *t* < 70), or clinical (*t* > 70). The C-TRF is designed to target preschool children ages 2 to 5.

#### 2.2.2. The Teacher Report Form (TRF)

The Teacher’s Report Form (TRF) measures the prevalence and intensity of clinical symptoms in children and adolescents (6–18 years). The TRF has 113 items; the teacher scores each item from 0 to 2 points, were: 0 = “not true”, 1 = “sometimes true”, and 2 = “very true”; higher scores indicate a higher symptom intensity. Raw scores are then converted to *t*-scores, which are classified as: normal (*t* < 65), risk (65 < *t* < 70), or clinical (*t* > 70). 

The items are categorized in two main scales, the DSM-oriented scale, and the Syndrome scale. The DSM-oriented domains include affective problems, anxiety problems, somatic problems, attention deficit and hyperactivity problems, oppositional defiant problems, and conduct problems. The internal consistency of the subscales ranges from 0.72 to 0. The Syndrome scale includes the following subscales: anxious/depressed, withdrawn/depressed, somatic complaints, social problems, thought problems, attention problems, rule-breaking behavior, aggressive behavior, and other problems; the internal consistency of such subscales range from 0.34 to 0.94, see Table 3. 

### 2.3. Data Analysis

After data was collected and digitalized, item scores were added according to their classification in either Syndrome or DSM-oriented scales. Internal consistency was obtained through Cronbach’s alpha (α), with 95% confidence intervals. These totals were converted to *t*-scores, which were classified as normal, risk, or clinical according to the ASEBA methodology. Absolute and relative frequencies were used to determine the prevalence of each subscale, 95% confidence intervals were built for each relative frequency. Prevalence data was determined separately for girls and boys. All analyses were made using SPSS version 26 and JASP version 0.14 [39]. 

### 2.4. Ethical Considerations

The current project was approved by the Regional Ministry of Education and Science, Provincial Delegation of Malaga, Regional Government of Andalusia, RG Fulfilling the following requirements: Request for authorization from the school management and school council where the pupils who are the object of the project attend school; authorization from the father/mother or guardian of each of the pupils, who have previously been informed of the purpose of the project, the methods to be used, and the use of the results; guarantee of the confidentiality of the nominal data obtained (L.O. 15/1999 of 13 December on the Protection of Personal Data).

## 3. Results

### 3.1. Results from the Caregiver-Teacher Report Form (C-TRF)

Overall, there was a low prevalence of clinical scores in the DSM-oriented scales, with attention deficit and hyperactivity problems having the highest number of children with clinical (*n* = 2; 1.13%) and at-risk scores (*n* = 10; 5.65%). A comparison of the 95% confidence intervals reveals no statistically significant difference in prevalence levels between boys and girls, see Table 4. 

The highest clinical in the C-TRF syndrome scales were found in the withdrawn (*n* = 3; 1.69%) and attention problem (*n* = 3; 1.69%) scores. Overall, clinical levels of externalizing scores (*n* = 8; 4.52%) were more prevalent than those of internalizing scores (*n* = 3; 1.69%). However, a confidence interval comparison reveals no statistically significant difference, see Table 5 Aggressive behaviors were the most prevalent at-risk scores (*n* = 8; 4.52%), being non-significantly more prevalent in boys (*n* = 6; 6.67%) than in girls (*n* = 2; 2.30%). Comparing the 95% confidence intervals reveals no statistically significant difference in prevalence levels between boys and girls. 

### 3.2. Results from the Teacher Report Form (TRF)

Overall, the DSM-oriented scales with the highest prevalence of children with clinical scores correspond to anxiety problems (*n* = 10; 4.12%). A significant number of children are at risk of suffering attention deficit and hyperactivity problems (*n* = 27; 11.11%) and affective problems (*n* = 25; 10.29%). For girls, affective problems (*n* = 5; 3.62%) are the most prevalent clinical scores, while attention deficit and hyperactivity problems (*n* = 18; 13.04%) and affective problems (*n* = 16; 11.59%) are the most prevalent risk scores. For boys, the anxiety problems subscale (*n* = 6; 5.71%) is the most prevalent clinical subscale, while affective problems (*n* = 9; 8.57%) and attention deficit and hyperactivity problems (*n* = 9; 8.57%) are the most prevalent risk scores. A comparison of the 95% confidence intervals reveals no statistically significant difference between boys and girls. Table 6 presents the statistical description of the scores obtained on the DSM-oriented scales.

A comparison between our findings and the prevalence data from Polanczyk et al. (2015) [32] shows that at a 95% confidence interval, there is no significant difference between the Spanish prevalence of mental health problems when compared to world data, see Table 7. 

Clinical levels of externalizing problems are found in 9.47% (*n* = 23) of the total sample, being more prevalent than internalizing problems (*n* = 16; 6.58%). Clinical levels of internalizing problems were non-significantly more frequent in boys (*n* = 10; 9.52%) than in girls (*n* = 6; 4.35%). A comparison of the 95% confidence intervals reveals that none of the syndrome scales differ significantly between boys and girls, see Table 8. 

## 4. Discussion

The present study’s findings emphasize the importance of the teacher as a key actor of school-based mental-health screenings. Our study suggests that internalizing problems are less prevalent than externalizing syndromes. Given that internalizing disorders implies internal distress instead of overt behaviors, such disorders may be hard to detect, mainly when children have not fully developed their communication skills. Internalizing disorders in children may be underestimated by parents and teachers, who might consider internalizing behaviors as less problematic than externalizing disorders [40]. The dimensional scores of the ASEBA methodology (internalizing and externalizing problems) may serve as an essential tool to assess possible individual treatments in children [30]. 

No statistically significant differences were found between boys and girls in any of the CTRF and TRF scales. This result is unexpected, as previous research has found significant sex-related differences in the development of specific externalizing behaviors [41]. Moreover, a notable result is the absence of somatic symptoms detected by the teachers. This could be because children with somatic symptoms may not attend school until they feel healthy. 

Recent research has found that children and adolescents that suffer internalizing and externalizing problems are at significantly higher risk of having work incapacities (sickness absence and disability pension) as adults. Such results suggest that early mental health prevention, detection, and interventions may have positive effects that persist through adulthood [42]. Therefore, educational detection systems are necessary to properly identify psychosocial problems in children, families, and their dynamics. Such early canalization may help prevent somatic complaints, which may continue from childhood to adolescence [43]. Therefore, interventions should also consider parents’ and caretakers’ mental health states as recent studies have shown that parenting stress is related to children’s level of internalizing and externalizing problems [44].

The prevalence of mental health problems has significantly increased in the last decades, particularly in children and adolescents within the school setting [45], with repercussions on life’s personal, social, economic, and academic domains [46]. The integration of mental health detection and intervention services within the school system, at the classroom and individual levels, is of prime importance to promote students’ well-being [18]. This has forced teachers to go beyond their traditional role and focus on their students’ mental health. Simultaneously, a continuous focus on students’ well-being can also have a detrimental impact on teachers’ mental health. Therefore, school systems should also consider mental health programs to promote the well-being of their staff [16]. 

To address students’ mental health needs, it is necessary to develop strategies that consider child development’s biological, psychological, and social dimensions [3,47]. This can be achieved through ecological-systemic interventions [48], where schools, families, and communities are considered key components of integral early child development [18,48]. Previous research has shown that universal health promotion and prevention programs within the school setting are highly beneficial for all ages [14,49,50,51]. The data obtained from school contexts is helpful in the design of public policies [19]. 

Positive school mental health screenings make it possible to identify personal, curricular, institutional, family, and community-related assets that can be used to design mental health promotion programs. This could be achieved through school-entrance screenings that allow for early identification of potential clinical problems and their corresponding intervention [52]. This is vital, as early detection and evidence-based interventions are essential for ensuring fair academic opportunities for all students [53]. 

Regarding such interventions, the development and effectiveness of Parent Training Programs based on Cognitive Behavioral Therapy (CBT) should be explored to promote children’s mental health. CBT has proven effective in minimizing internalizing and externalizing disorders in children and adolescents [54,55]. Multimodal approaches involving children, parents, and other actors, are particularly effective when combined with CBT to address externalizing problems [55]. 

This requires adequate staff training in mental health issues and evidence-based interventions, as well as constant monitoring [56]. However, many teachers feel they have not received proper training for such work [57]. School-based mental health programs should be interconnected with external health care systems, and they should also promote student and family engagement. This requires proper staff training in mental health-related topics and interventions based on evidence and constant supervision [56]. Common teacher-based interventions include social skills training, cognitive-behavioral approaches, or a combination of these. For the most part, such intervention benefits from the significant amount of time teachers and students share [58]. 

We believe that dimensional, universal, and broad-spectrum screening instruments, such as the C-TRF and the TRF, are ideal tools that can be used to detect students at-risk within the school settings. Even more so when considering that teachers are a key component in detecting students’ mental health indicators. This evaluation model results in a less invasive and more viable approach that might be easily incorporated into the school structure by reaching and benefitting all students [18]. 

Despite the practical value of our findings, the current research is not without limitations. For instance, the non-probabilistic nature of the sample may limit its representativeness and, therefore, the generalizability of the results. The small sample size in clinical levels may reduce the precision of the confidence intervals. On the other hand, given the importance of cross-culturally validated mental health assessment instruments in non-English languages [24], future research should validate the C-TRF and TRF use in the Spanish population. This is of particular relevance when considering the multilingual and multicultural background of the Spanish population. In this sense, low reliability in the C-TRF should also be considered. 

Additionally, the C-TRF and the TRF are instruments designed to measure psychopathology. However, the current notion of mental health also involves the positive conditions and emotions of human existence. Future research should focus on positive school mental health strategies. Regarding this, students’ social climate, peer relationships, and optimism should be considered in potential studies [59]. On another point, considering our data was collected before the COVID-19 pandemic, future research should focus on students’ mental health in the post-pandemic setting. 

Another limitation is that the C-TRF and TRF are based on teachers’ observational reports in the school context. More information is needed to understand and triangulate children’s behavior in other settings, such as within the familiar context. Finally, the instruments presented here are used as part of a screening tool and do not constitute, by itself, a clinical diagnosis. 

## 5. Conclusions

School-based detection and intervention are key components to assure positive mental health in children, with teachers playing an essential role in assessing students’ well-being. In 5-year-old children, the DSM-oriented scale with the highest clinical prevalence corresponds to attention deficit and hyperactivity problems (1.13%). In this same sub-sample, clinical levels of externalizing problems (4.52%) were non-significantly more common than internalizing conditions (1.69%). As for children between 6 and 8 years old, the DSM-oriented scale with the highest prevalence of clinical scores corresponds to anxiety problems (4.12%) and conduct problems (2.88%). Again, clinical levels of externalizing problems (9.47%) were non-significantly more prevalent than internalizing problems (6.58%). The results present 95% prevalence confidence intervals in the general population and sex-differentiated descriptive statistics. 

Consequently, our findings should be shared with the community, including parents, teachers, school board members, public authorities, and community mental health providers [60]. This could help integrate the systemic and multidisciplinary approaches necessary to provide sustainable mental health interventions within a school and community-engaged setting [38]. We recommend the normalized use of the ASEBA methodology as a screening tool for school-based interventions. 

## Figures and Tables

**Table 1:** Characteristics of the sample.

Descriptor	Total		C-TRF		TRF	
	*n*	%	*n*	%	*n*	%
Sex						
Male	195	46.43%	90	50.80%	105	43.20%
Female	225	53.57%	87	49.20%	138	56.80%
Age						
5 years	177	42.14%	177	100%	-	-
6 years	129	30.71%	-	-	129	53.10%
7 years	92	21.90%	-	-	92	37.90%
8 years	22	5.24%	-	-	22	9.10%
School						
El Albero	55	13.10%	55	31.10%	-	-
Azahar	11	2.62%	11	6.20%	-	-
Los Boliches	62	14.76%	19	10.70%	43	17.70%
Sohail	89	21.19%	24	13.60%	65	26.70%
Andalucía	84	20.00%	31	17.50%	53	21.80%
Valdelecrín	54	12.86%	8	4.50%	46	18.90%
Cervantes	65	15.48%	29	16.40%	36	14.80%
Grade						
Pre-school	177	42.14%	177	100.00%	-	-
First grade	141	33.57%	-	-	141	58.00%
Second grade	102	24.29%	-	-	102	42.00%
Total ^a^	420	10.00%	177	42.14%	243	57.86%

Note. ^a^ The percentage represents row totals.

**Table 2:** Internal consistency for the C-TRF.

Scale	Sub-Scale	Cronbach’s *α*	*LL*	*UL*	Number of Items
DSM-oriented	Affective Problems	0.74	0.68	0.80	7
	Anxiety Problems	0.65	0.57	0.72	7
	Pervasive Developmental Problems	0.76	0.70	0.81	13
	Attention Deficit and Hyperactivity Problems	0.92	0.90	0.93	13
	Oppositional Defiant Problems	0.81	0.77	0.85	7
Syndrome	Emotionally Reactive	0.72	0.66	0.78	7
	Anxious & Depressed	0.65	0.57	0.73	8
	Somatic Complaints	0.70	0.63	0.76	7
	Withdrawn	0.83	0.78	0.86	10
	Attention Problems	0.86	0.82	0.89	9
	Aggressive Behavior	0.94	0.93	0.95	25
	Other Problems	0.80	0.76	0.84	34

Note. Intervals are built at a 95% confidence level, were: *LL* = Lower Limit and *UL* = Upper Limit.

**Table 3:** Internal consistency for the TRF.

Scale	Sub-Scale	Cronbach’s *α*	*LL*	*UL*	Number of Items
DSM-oriented	Affective Problems	0.73	0.67	0.79	10
	Anxiety Problems	0.72	0.64	0.79	6
	Somatic Problems	-	-	-	7
	Attention Deficit and Hyperactivity Problems	0.92	0.90	0.94	13
	Oppositional Defiant Problems	0.79	0.73	0.84	6
	Conduct Problems	0.85	0.81	0.88	13
Syndrome	Anxious/Depressed	0.77	0.71	0.82	16
	Withdrawn/Depressed	0.79	0.73	0.83	8
	Somatic Complaints	0.34	0.19	0.48	9
	Social Problems	0.71	0.66	0.76	11
	Thought Problems	0.72	0.66	0.77	10
	Attention Problems	0.94	0.93	0.95	26
	Rule-Breaking Behavior	0.71	0.65	0.76	12
	Aggressive Behavior	0.92	0.91	0.94	20
	Other Problems	0.42	0.30	0.52	8

Note. Intervals are calculated at a confidence level of 95%, where: *LL* = Lower Limit and *UL* = Upper Limit. No information is available regarding the reliability of the somatic problems subscale as all associated items were scored as “0”.

**Table 4:** Prevalence of DSM-oriented problems found through the C-TRF.

Scale	Level	General				Girls				Boys			
		*n*	%	% *LL*	% *UL*	*n*	%	% *LL*	% *UL*	*n*	%	% *LL*	% *UL*
1	Normal	169	95.48	91.29	98.03	84	96.55	90.25	99.28	85	94.44	87.51	98.17
	Risk	7	3.95	1.6	7.98	2	2.3	0.28	8.06	5	5.56	1.83	12.49
	Clinical	1	0.56	0.01	3.11	1	1.15	0.03	6.24	0	0	0	4.02
2	Normal	174	98.31	95.13	99.65	86	98.85	93.76	99.97	88	97.78	92.2	99.73
	Risk	2	1.13	0.14	4.02	0	0	0	4.15	2	2.22	0.27	7.8
	Clinical	1	0.56	0.01	3.11	1	1.15	0.03	6.24	0	0	0	4.02
3	Normal	173	97.74	94.32	99.38	85	97.7	91.94	99.72	88	97.78	92.2	99.73
	Risk	3	1.69	0.35	4.87	1	1.15	0.03	6.24	2	2.22	0.27	7.8
	Clinical	1	0.56	0.01	3.11	1	1.15	0.03	6.24	0	0	0	4.02
4	Normal	165	93.22	88.46	96.45	82	94.25	87.1	98.11	83	92.22	84.63	96.82
	Risk	10	5.65	2.74	10.14	4	4.6	1.27	11.36	6	6.67	2.49	13.95
	Clinical	2	1.13	0.14	4.02	1	1.15	0.03	6.24	1	1.11	0.03	6.04
5	Normal	173	97.74	94.32	99.38	86	98.85	93.76	99.97	87	96.67	90.57	99.31
	Risk	3	1.69	0.35	4.87	0	0	0	4.15	3	3.33	0.69	9.43
	Clinical	1	0.56	0.01	3.11	1	1.15	0.03	6.24	0	0	0	4.02

Note. 1 = Affective problems, 2 = anxiety problems, 3 = pervasive developmental problems, 4 = attention deficit and hyperactivity problems, 5 = oppositional defiant problems. Intervals are calculated at a confidence level of 95%, where: *LL* = Lower Limit and *UL* = Upper Limit.

**Table 5:** Prevalence of syndrome scales of the C-TRF.

Scale	Level	General				Girls				Boys			
		*n*	%	% *LL*	% *UL*	*n*	%	% *LL*	% *UL*	*n*	%	% *LL*	% *UL*
1	Normal	171	96.61	92.77	98.75	86	98.85	93.76	99.97	85	94.44	87.51	98.17
	Risk	5	2.82	0.92	6.47	0	0	0	4.15	5	5.56	1.83	12.49
	Clinical	1	0.56	0.01	3.11	1	1.15	0.03	6.24	0	0	0	4.02
2	Normal	173	97.74	94.32	99.38	85	97.7	91.94	99.72	88	97.78	92.2	99.73
	Risk	2	1.13	0.14	4.02	0	0	0	4.15	2	2.22	0.27	7.8
	Clinical	2	1.13	0.14	4.02	2	2.3	0.28	8.06	0	0	0	4.02
3	Normal	174	98.31	95.13	99.65	86	98.85	93.76	99.97	88	97.78	92.2	99.73
	Risk	2	1.13	0.14	4.02	0	0	0	4.15	2	2.22	0.27	7.8
	Clinical	1	0.56	0.01	3.11	1	1.15	0.03	6.24	0	0	0	4.02
4	Normal	173	97.74	94.32	99.38	85	97.7	91.94	99.72	88	97.78	92.2	99.73
	Risk	1	0.56	0.01	3.11	1	1.15	0.03	6.24	0	0	0	4.02
	Clinical	3	1.69	0.35	4.87	1	1.15	0.03	6.24	2	2.22	0.27	7.8
5	Normal	171	96.61	92.77	98.75	86	98.85	93.76	99.97	85	94.44	87.51	98.17
	Risk	3	1.69	0.35	4.87	0	0	0	4.15	3	3.33	0.69	9.43
	Clinical	3	1.69	0.35	4.87	1	1.15	0.03	6.24	2	2.22	0.27	7.8
6	Normal	168	94.92	90.57	97.65	84	96.55	90.25	99.28	84	93.33	86.05	97.51
	Risk	8	4.52	1.97	8.71	2	2.3	0.28	8.06	6	6.67	2.49	13.95
	Clinical	1	0.56	0.01	3.11	1	1.15	0.03	6.24	0	0	0	4.02
7	Normal	166	93.79	89.15	96.86	84	96.55	90.25	99.28	82	91.11	83.23	96.08
	Risk	8	4.52	1.97	8.71	0	0	0	4.15	8	8.89	3.92	16.77
	Clinical	3	1.69	0.35	4.87	3	3.45	0.72	9.75	0	0	0	4.02
8	Normal	161	90.96	85.74	94.74	82	94.25	87.1	98.11	79	87.78	79.18	93.74
	Risk	8	4.52	1.97	8.71	2	2.3	0.28	8.06	6	6.67	2.49	13.95
	Clinical	8	4.52	1.97	8.71	3	3.45	0.72	9.75	5	5.56	1.83	12.49
9	Normal	162	91.53	86.41	95.18	83	95.4	88.64	98.73	79	87.78	79.18	93.74
	Risk	8	4.52	1.97	8.71	0	0	0	4.15	8	8.89	3.92	16.77
	Clinical	7	3.95	1.6	7.98	4	4.6	1.27	11.36	3	3.33	0.69	9.43

Note. 1 = Emotionally reactive, 2 = anxious/depressed, 3 = somatic complaints; 4 = withdrawn, 5 = attention problems, 6 = aggressive behavior, 7 = internalizing, 8 = externalizing, 9 = total problems. Intervals are calculated at a confidence level of 95%, where: *LL* = Lower Limit and *UL* = Upper Limit.

**Table 6:** Prevalence of DSM-oriented problems using the TRF.

Scales	Level	General				Girls				Boys			
		*n*	%	% *LL*	% *UL*	*n*	%	% *LL*	% *UL*	*n*	%	% *LL*	% *UL*
1	Normal	212	87.24	82.38	91.16	117	84.78	77.68	90.33	95	90.48	83.18	95.34
	Risk	25	10.29	6.77	14.81	16	11.59	6.77	18.14	9	8.57	3.99	15.65
	Clinical	6	2.47	0.91	5.3	5	3.62	1.19	8.25	1	0.95	0.02	5.19
2	Normal	221	90.95	86.61	94.24	128	92.75	87.08	96.47	93	88.57	80.89	93.95
	Risk	12	4.94	2.58	8.47	6	4.35	1.61	9.22	6	5.71	2.13	12.02
	Clinical	10	4.12	1.99	7.44	4	2.9	0.8	7.26	6	5.71	2.13	12.02
3	Normal	243	100	98.49	100	138	100	97.36	100	105	100	96.55	100
	Risk	0	0	0	1.51	0	0	0	2.64	0	0	0	3.45
	Clinical	0	0	0	1.51	0	0	0	2.64	0	0	0	3.45
4	Normal	210	86.42	81.46	90.46	116	84.06	76.86	89.73	94	89.52	82.03	94.65
	Risk	27	11.11	7.45	15.75	18	13.04	7.92	19.83	9	8.57	3.99	15.65
	Clinical	6	2.47	0.91	5.3	4	2.9	0.8	7.26	2	1.9	0.23	6.71
5	Normal	230	94.65	91.03	97.12	129	93.48	87.98	96.97	101	96.19	90.53	98.95
	Risk	11	4.53	2.28	7.96	8	5.8	2.54	11.1	3	2.86	0.59	8.12
	Clinical	2	0.82	0.1	2.94	1	0.72	0.02	3.97	1	0.95	0.02	5.19
6	Normal	227	93.42	89.53	96.19	128	92.75	87.08	96.47	99	94.29	87.98	97.87
	Risk	9	3.7	1.71	6.91	7	5.07	2.06	10.17	2	1.9	0.23	6.71
	Clinical	7	2.88	1.17	5.84	3	2.17	0.45	6.22	4	3.81	1.05	9.47

Note. 1 = Affective problems, 2 = anxiety problems, 3 = somatic problems; 4 = attention deficit and hyperactivity problems, 5 = oppositional defiant problems, 6 = conduct problems. Intervals are calculated at a confidence level of 95%, where: *LL* = Lower Limit and *UL* = Upper Limit.

**Table 7:** A comparison of the prevalence of mental health indicators between the current Spanish sample and world prevalence.

Disorder	The Present Study			World Data (Polanczyk et al., 2015)		
	%	*LL*	*UL*	%	*LL*	*UL*
Anxiety	4.12	1.99	7.44	6.5	4.7	9.1
Depressive disorder	2.47	0.91	5.3	3.4	2.6	4.5
Disruptive disorder	2.88	1.17	5.84	5.7	4	8.1

Note. Depressive disorders of Polanczyk et al. (2015) [32] are compared to the TRF DSM-oriented scale of Affective Problems; Disruptive disorders are compared to the TRF DSM-oriented scale of conduct problems. Intervals are presented at 95% confidence.

**Table 8:** Prevalence of syndromes according to the TRF.

Scales	Level	General				Girls				Boys			
		*n*	%	% *LL*	% *UL*	*n*	%	% *LL*	% *UL*	*n*	%	% *LL*	% *UL*
1	Normal	231	95.06	91.53	97.42	132	95.65	90.78	98.39	99	94.29	87.98	97.87
	Risk	4	1.65	0.45	4.16	2	1.45	0.18	5.14	2	1.9	0.23	6.71
	Clinical	8	3.29	1.43	6.38	4	2.9	0.8	7.26	4	3.81	1.05	9.47
2	Normal	226	93	89.04	95.87	131	94.93	89.83	97.94	95	90.48	83.18	95.34
	Risk	14	5.76	3.19	9.48	6	4.35	1.61	9.22	8	7.62	3.35	14.46
	Clinical	3	1.23	0.26	3.57	1	0.72	0.02	3.97	2	1.9	0.23	6.71
3	Normal	236	97.12	94.16	98.83	135	97.83	93.78	99.55	101	96.19	90.53	98.95
	Risk	7	2.88	1.17	5.84	3	2.17	0.45	6.22	4	3.81	1.05	9.47
	Clinical	0	0	0	1.51	0	0	0	2.64	0	0	0	3.45
4	Normal	229	94.24	90.52	96.81	129	93.48	87.98	96.97	100	95.24	89.24	98.44
	Risk	9	3.7	1.71	6.91	6	4.35	1.61	9.22	3	2.86	0.59	8.12
	Clinical	5	2.06	0.67	4.74	3	2.17	0.45	6.22	2	1.9	0.23	6.71
5	Normal	239	98.35	95.84	99.55	136	98.55	94.86	99.82	103	98.1	93.29	99.77
	Risk	2	0.82	0.1	2.94	1	0.72	0.02	3.97	1	0.95	0.02	5.19
	Clinical	2	0.82	0.1	2.94	1	0.72	0.02	3.97	1	0.95	0.02	5.19
6	Normal	231	95.06	91.53	97.42	134	97.1	92.74	99.2	97	92.38	85.54	96.65
	Risk	11	4.53	2.28	7.96	4	2.9	0.8	7.26	7	6.67	2.72	13.25
	Clinical	1	0.41	0.01	2.27	0	0	0	2.64	1	0.95	0.02	5.19
7	Normal	233	95.88	92.56	98.01	135	97.83	93.78	99.55	98	93.33	86.75	97.28
	Risk	9	3.7	1.71	6.91	3	2.17	0.45	6.22	6	5.71	2.13	12.02
	Clinical	1	0.41	0.01	2.27	0	0	0	2.64	1	0.95	0.02	5.19
8	Normal	228	93.83	90.02	96.5	132	95.65	90.78	98.39	96	91.43	84.35	96.01
	Risk	11	4.53	2.28	7.96	5	3.62	1.19	8.25	6	5.71	2.13	12.02
	Clinical	4	1.65	0.45	4.16	1	0.72	0.02	3.97	3	2.86	0.59	8.12
9	Normal	207	85.19	80.08	89.4	119	86.23	79.34	91.5	88	83.81	75.35	90.28
	Risk	20	8.23	5.1	12.43	13	9.42	5.11	15.57	7	6.67	2.72	13.25
	Clinical	16	6.58	3.81	10.47	6	4.35	1.61	9.22	10	9.52	4.66	16.82
10	Normal	206	84.77	79.63	89.05	115	83.33	76.05	89.13	91	86.67	78.64	92.51
	Risk	14	5.76	3.19	9.48	10	7.25	3.53	12.92	4	3.81	1.05	9.47
	Clinical	23	9.47	6.09	13.86	13	9.42	5.11	15.57	10	9.52	4.66	16.82
11	Normal	235	96.71	93.62	98.57	135	97.83	93.78	99.55	100	95.24	89.24	98.44
	Risk	7	2.88	1.17	5.84	3	2.17	0.45	6.22	4	3.81	1.05	9.47
	Clinical	1	0.41	0.01	2.27	0	0	0	2.64	1	0.95	0.02	5.19

Note. 1 = Anxious and depressed, 2 = withdrawn and depressed, 3 = somatic complaints, 4 = social problems, 5 = thought problems, 6 = attentional problems, 7 = rule-breaking behavior, 8 = aggressive behavior, 9 = internalizing, 10 = externalizing, 11 = total problems. Intervals are calculated at a confidence level of 95%, where: *LL* = Lower Limit and *UL* = Upper Limit.

## Data Availability

The data that support the findings of this study are available from the corresponding author, ACR, upon reasonable request.
